# Mitochondria-Associated Membrane Dysfunction in Neurodegeneration and Its Effects on Lipid Metabolism, Calcium Signaling, and Cell Fate

**DOI:** 10.3390/membranes15090263

**Published:** 2025-08-31

**Authors:** Thi Thuy Truong, Alka Ashok Singh, Nguyen Van Bang, Nguyen Minh Hung Vu, Sungsoo Na, Jaeyeop Choi, Junghwan Oh, Sudip Mondal

**Affiliations:** 1 Industry 4.0 Convergence Bionics Engineering, Department of Biomedical Engineering, Pukyong National University, Busan 48513, Republic of Korea; truongthuy@pukyong.ac.kr (T.T.T.); bangnv_pknu@pukyong.ac.kr (N.V.B.); vnmhung@pukyong.ac.kr (N.M.H.V.); 2 Department of Life Sciences, Yeungnam University, Gyeongsan 38541, Republic of Korea; alkasingh10f@yu.ac.kr; 3 Weldon School of Biomedical Engineering, Purdue University, Indianapolis, IN 46202, USA; na19@purdue.edu; 4 Smart Gym-Based Translational Research Center for Active Senior’s Healthcare, Pukyong National University, Busan 48513, Republic of Korea; jaeyeopchoi@pknu.ac.kr; 5 Ohlabs Corp., Busan 48513, Republic of Korea; 6 Digital Healthcare Research Center, Institute of Information Technology and Convergence, Pukyong National University, Busan 48513, Republic of Korea

**Keywords:** mitochondria-associated membranes (MAMs), endoplasmic reticulum–mitochondria tethering, lipid metabolism, calcium homeostasis, neurodegeneration, apoptosis, autophagy

## Abstract

Mitochondria-associated membranes (MAMs) are essential for cellular homeostasis. MAMs are specialized contact sites located between the endoplasmic reticulum (ER) and mitochondria and control apoptotic pathways, lipid metabolism, autophagy initiation, and calcium signaling, processes critical to the survival and function of neurons. Although this area of membrane biology remains understudied, increasing evidence links MAM dysfunction to the etiology of major neurodegenerative diseases, such as Alzheimer’s disease, Parkinson’s disease, and amyotrophic lateral sclerosis (ALS). MAMs consist of a network of protein complexes that mediate molecular exchange and ER–mitochondria tethering. MAMs regulate lipid flow in the brain, including phosphatidylserine and cholesterol; disruption of this process causes membrane instability and impaired synaptic function. Inositol 1,4,5-trisphosphate receptor—voltage-dependent anion channel 1 (IP3R-VDAC1) interactions at MAMs maintain calcium homeostasis, which is required for mitochondria to produce ATP; dysregulation promotes oxidative stress and neuronal death. An effective therapeutic approach for altering neurodegenerative processes is to restore the functional integrity of MAMs. Improving cell-to-cell interactions and modulating MAM-associated proteins may contribute to the restoration of calcium homeostasis and lipid metabolism, both of which are key for neuronal protection. MAMs significantly contribute to the progression of neurodegenerative diseases, making them promising targets for future therapeutic research. This review emphasizes the increasing importance of MAMs in the study of neurodegeneration and their potential as novel targets for membrane-based therapeutic interventions.

## 1. Introduction

Neurodegenerative diseases such as Alzheimer’s disease (AD), Parkinson’s disease (PD), ALS, and Huntington’s disease (HD) represent a growing public health concern worldwide, marked by the progressive loss of neuronal structure and function [1]. The progressive loss of anatomically or physiologically significant brain systems is a characteristic observed in a wide range of disorders [2]. Despite their diverse clinical manifestations and etiologies, these disorders share common pathological features, including mitochondrial dysfunction, oxidative stress, defective autophagy, and impaired calcium homeostasis [3]. Recent studies have highlighted the critical role of inter-organelle communication, particularly between the endoplasmic reticulum (ER) and mitochondria, in orchestrating cellular homeostasis and neuronal survival [4]. Mitochondria are crucial for energy production in cells. Defects in the mitochondria and the resulting symptoms stem from mitochondrial disorders [3]. The structural and functional interface that facilitates this communication is known as the mitochondria-associated membrane (MAM), a specialized subdomain of the ER intimately connected to the outer mitochondrial membrane. The ER and mitochondria are physiologically regulated by MAMs, which preserve calcium signaling and mitochondrial biogenesis [5], and serve as signaling and metabolic hubs that mediate several essential cellular processes. MAMs contain thousands of proteins, and their roles vary significantly, with some proteins acting as anchors [6]. MAMs play a crucial role in lipid synthesis, Ca^2+^ homeostasis regulation, ER–mitochondrial function coordination, and the communication of death signals between the ER and mitochondria [7]. Structurally, they are composed of protein complexes that tether the ER and mitochondria at close proximity (10–30 nm), allowing efficient exchange of metabolites and signals [8]. Vesicle-associated membrane protein-associated protein B (VAPB) is an integral protein in the ER membrane and is implicated in the ER unfolded protein response and the regulation of cellular calcium homeostasis [9]. VAPB has been reported to interact with the mitochondrial outer membrane protein tyrosine phosphatase-interacting protein 51 (PTPIP51) [10]. Disruption of these complexes alters ER–mitochondria communication, contributing to cellular dysfunction and neurodegeneration.

In the context of neurodegenerative diseases, mounting evidence highlights the dysregulation of MAM-resident proteins and signaling pathways. For instance, presenilin-1 and presenilin-2, implicated in familial AD, are enriched in MAMs and modulate calcium signaling and lipid homeostasis [11]. Mutations in presenilin-1 (PS1) and -2 (PS2) cause human familial forms of AD. Presenilins are present in many compartments in the cell, including the ER and mitochondria [12]. Similarly, alpha-synuclein, whose aggregation is central to PD pathogenesis, has been shown to localize at MAMs and interfere with ER–mitochondria tethering [13]. The abnormal accumulation of mutant proteins such as SOD1 in ALS [14] and huntingtin in HD further disrupts MAM function, exacerbating mitochondrial stress, reactive oxygen species (ROS) generation, and impaired energy metabolism [15]. These disruptions initiate a cascade of pathological events that culminate in synaptic dysfunction, neuronal death, and brain atrophy.

Given the central role of MAMs in coordinating calcium dynamics and lipid metabolism, two processes critical for neuronal viability, understanding their dysfunction offers a unifying perspective in neurodegeneration research [16]. Particularly, the abnormal lipid profiles observed in several neurodegenerative conditions may result from impaired lipid biosynthesis or trafficking at the MAM [17]. Similarly, dysregulated calcium flux between the ER and mitochondria can lead to mitochondrial calcium overload, ROS production, and activation of cell death pathways [18]. Therefore, the MAM not only represents a site of early pathological changes but also offers potential targets for therapeutic intervention aimed at restoring organelle communication and preventing neuronal loss [19].

**Figure 1:**
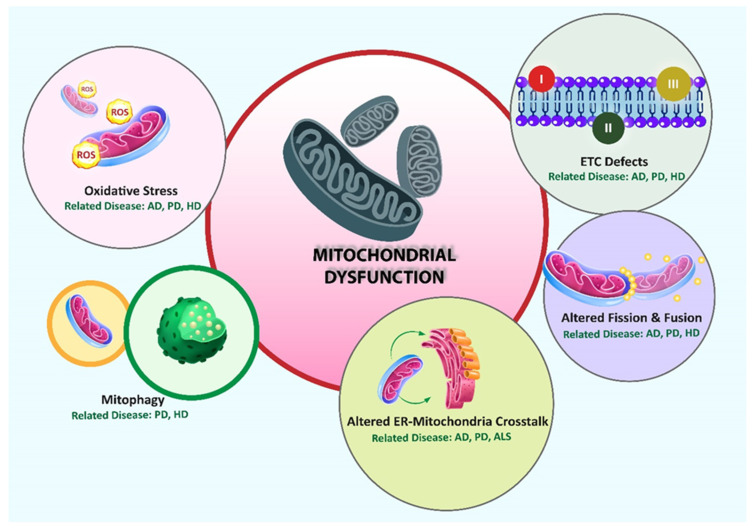
The six domains of the mitochondria-associated membranes. Figure 1 summarizes the multifunctional domains of MAMs in neurodegeneration. The six domains of the mitochondria-associated membranes (MAMs)—autophagy, apoptosis, inflammation, lipid metabolism, phosphatidylserine (PtdSer) synthesis, and calcium homeostasis—are highlighted in the schematic. All domains involve alpha-synuclein (αSyn). Interactions between the ER and mitochondria are anchored by four main MAM tethers: inositol 1,4,5-trisphosphate receptor (IP3R)–GRP75–VDAC, mitofusin-2 (MFN2), BAP31–FIS1, and VAPB–PTPIP51. MAM plays an important role in the pathophysiology of Parkinson’s disease (PD), as evidenced by the presence of several proteins linked to the disease, such as Parkin, PINK1, DJ-1, LRRK2, MIRO, 14-3-3, and VPS13. MAMs act as key hubs for the mechanisms underlying neurodegenerative diseases, as these proteins support essential cellular functions such as calcium signaling, lipid transfer, autophagosome formation, and stress responses [13]. Copyrighted and modified by the author and Licensee MDPI, Basel, Switzerland, distributed under Creative Commons Attribution (CC BY) license (https://creativecommons.org/licenses/by/4.0/). https://doi.org/10.3390/ijms25126525, under CC BY 4.0.

This review aims to provide a comprehensive overview of the structural and functional characteristics of MAMs and their role in the pathophysiology of neurodegenerative diseases. We critically examine the impact of MAM dysfunction on lipid metabolism, which disrupts calcium signaling and regulates autophagy and apoptosis, thereby influencing neuronal fate. Furthermore, we highlight emerging therapeutic strategies that target MAM components to restore cellular homeostasis. By integrating insights from diverse neurodegenerative models, this review seeks to establish MAMs as a central platform for understanding and treating neurodegenerative diseases. Several studies suggest that MAM domains are linked to a variety of biochemical pathways, and that key regulatory proteins needed for many cellular processes are either enriched or localized at MAMs (Figure 1) [13].

## 2. Structure and Composition of Mitochondria-Associated Membranes (MAMs)

The ER and mitochondria are essential organelles that influence several physiological and pathological cellular functions. Recent studies suggest that between 5% and 20% of the outer mitochondrial membrane can physically link with the ER in a highly dynamic manner, while maintaining a distance of approximately 10–30 nm. In eukaryotic cells, these connections, known as MAMs, represent a comparatively conserved structure that serves as a vital platform for material exchange between the ER and mitochondria, helping preserve several facets of cellular homeostasis. Particularly, the structure and function of MAMs are closely linked to ER-mediated Ca^2+^ release and recycling [20]. Far from being passive structural links, MAMs serve as key platforms for the integration of metabolic and signaling pathways critical to cellular survival, particularly in neurons, where energy demand and signal regulation are tightly coupled [13]. In addition to neurons, glial cells such as astrocytes show MAM activity, which contributes to calcium buffering, lipid metabolism, and neuroinflammatory responses, though their roles are less explored.

### 2.1. MAM Architecture and Tethering Proteins

The structural integrity of MAMs depends on a set of tethering proteins that physically bridge the outer mitochondrial membrane (OMM) and the ER membrane. MAMs contain thousands of proteins, and their roles vary significantly. Some tethering proteins help maintain MAMs by anchoring them [6]. According to the study, tethering proteins must possess particular features. Tethering proteins may be (1) proteins or protein complexes that directly participate in the physical connection between the ER and mitochondria or (2) interfering proteins or protein complexes that can directly influence the width of the gap, the area of contact, or the number of contact sites between the ER and mitochondria [7]. One of the best-characterized tethering complexes, mitofusin-2 (MFN2), is a GTPase localized on both the ER and mitochondrial membranes [21]. Although MFN2 was initially considered a positive tether, later studies suggest that it may act as a negative regulator of ER–mitochondria juxtaposition, depending on the cellular context [22]. The VAPB-PTPIP51 complex represents another critical tethering system. The fundamental architecture of MAMs has been extensively studied, with the vesicle-associated membrane protein-associated protein-B (VAPB)–protein tyrosine phosphatase-interacting protein-51 (PTPIP51) tether being critical in maintaining MAM integrity [23,24]. VAPB (vesicle-associated membrane protein-associated protein B) is located on the ER membrane, whereas PTPIP51 (protein tyrosine phosphatase-interacting protein 51) is anchored in the mitochondrial membrane. Their interaction modulates calcium signaling and autophagy by regulating the distance and communication between the two organelles [24,25].

Another important protein in MAMs is the inositol 1,4,5-trisphosphate receptor (IP3R). The ER membrane contains three isoforms of IP3R, which physically interact with the voltage-dependent anion channel 1 (VDAC1) located on the outer mitochondrial membrane. This interaction is promoted by the molecular chaperone glucose-regulated protein 75 (GRP75). The IP3R-VDAC interaction is critical for the transport and regulation of Ca^2+^ between the ER and the mitochondria [26]. The dynamic regulation of these tethers determines not only the physical extent of MAMs but also their functional properties in different cellular states.

### 2.2. Lipid and Protein Composition of MAMs

**Figure 2:**
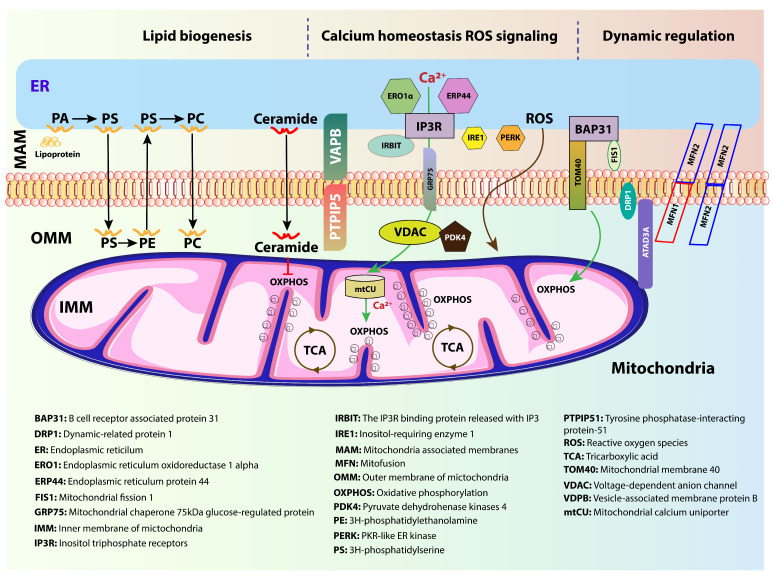
Schematic representation of the mitochondria-associated membrane (MAM), illustrating key protein complexes and lipid exchange mechanisms between the endoplasmic reticulum (ER) and mitochondria. The ER is depicted in green and the mitochondrion in orange, with the MAM region highlighted between them. Key proteins such as VDAC (voltage-dependent anion channel), GRP75, IP3R, ERP44, IRBIT, and ERO1α are labeled, representing essential components of the tethering and signaling machinery. Lipid transfer routes are indicated by blue arrows, showing the movement of phosphatidylserine (PS) from the ER to the mitochondria, where it is converted into phosphatidylethanolamine (PE). This diagram highlights the crucial role of MAMs in coordinating inter-organelle communication, calcium signaling, and lipid metabolism.

MAMs are uniquely enriched in specific lipids and proteins that distinguish them from the bulk ER and mitochondrial membranes [27]. In terms of lipid composition, MAMs serve as hotspots for the synthesis and transfer of phospholipids, such as phosphatidylserine (PS), which is synthesized in the ER and transferred to mitochondria, where it is converted into phosphatidylethanolamine (PE) [28]. Phospholipids are typically synthesized in the ER and then translocated to the membranes of other organelles, such as mitochondria. Phosphatidylcholine (PC) and PE are converted into PS in the ER by the MAM-resident enzymes PSS1 and PSS2. Following translocation to the mitochondria, PS is converted into PE by PS decarboxylase (PSD). The PE produced in the mitochondria is transported to the ER, where it is methylated by PEMT2 to form PC [29,30,31,32,33]. This lipid exchange is vital for maintaining mitochondrial membrane integrity and bioenergetics [34]. Additionally, cholesterol, ceramides, and sphingolipids are actively trafficked through MAMs [35], playing essential roles in regulating membrane fluidity, signaling, and apoptotic regulation (Figure 2).

The protein composition of MAMs is equally specialized and functionally diverse [36]. Proteomic studies have identified hundreds of proteins that transiently or stably associate with MAMs, reflecting their complexity and multifunctionality [37]. The dynamic assembly and disassembly of these protein complexes allow MAMs to respond rapidly to physiological changes. They coordinate vital functions such as calcium signaling, lipid metabolism, and cellular stress responses by acting as dynamic communication hubs between the ER and mitochondria. MAM malfunction has been recently linked to a variety of illnesses, including metabolic and neurodegenerative diseases [34].

### 2.3. Evolutionary and Cell-Type-Specific Variation

MAMs are evolutionarily conserved structures found across diverse eukaryotic lineages, highlighting their fundamental role in cellular physiology [38,39]. However, the composition and functional importance of MAMs can vary significantly across cell types and organisms [40]. For example, neurons and cardiomyocytes exhibit more extensive and tightly regulated ER–mitochondria contacts owing to their high metabolic demand and calcium flux requirements [41]. In contrast, MAMs in hepatocytes and adipocytes are more prominently involved in lipid metabolism and storage [42].

During development or in response to stress, the molecular profile of MAMs can undergo significant remodeling [43]. Certain diseases, including neurodegenerative disorders and metabolic syndromes, are associated with either excessive contact or loss of MAMs, suggesting that tight regulation of MAM architecture is critical for cellular health. Evolutionarily, divergence in MAM composition and functionality may reflect adaptations to specific energy demands, signaling mechanisms, and organelle architectures in different tissues [44].

## 3. MAM Dysfunction in Neurodegenerative Diseases

Studies have found links between MAMs and a variety of disorders, including neurological diseases, cancer, viral infections, obesity, and diabetes. Recent studies have linked MAM disruption to AD, PD, HD, ALS, and other neurodegenerative conditions [45] (Table 1). Disruption of MAMs is increasingly recognized as a converging pathological mechanism in major neurodegenerative diseases [46]. Given that MAMs orchestrate critical processes such as lipid metabolism, calcium signaling, and mitochondrial function, their dysfunction contributes to neuronal stress, synaptic failure, and cell death. This section highlights how MAM impairment manifests in four major neurodegenerative disorders—AD, PD, ALS, and HD—emphasizing the shared and disease-specific alterations [45,47]. MAMs emerge as a common link across these neurodegenerative illnesses, where pathogenic changes in calcium signaling, lipid metabolism, and mitochondrial dynamics intersect. MAM malfunction contributes to neuronal mortality and disease progression, whether through increased tethering (e.g., in AD), reduced connectivity (e.g., in PD and ALS), or structural interference by mutant proteins (e.g., in HD). Understanding these disease-specific changes provides new insights into MAMs as both biomarkers and therapeutic targets for neurodegeneration.

### 3.1. Alzheimer’s Disease (AD)

In recent years, there has been increased emphasis on understanding the contribution of MAMs to human pathophysiology, in general, and neurodegenerative disorders in particular. In the case of AD, numerous documented changes in mitochondrial behavior and function have been observed, including decreased respiratory chain activity and oxidative phosphorylation, increased free radical production, and altered organellar morphology, dynamics, and positioning (particularly perinuclear mitochondria) [48]. Furthermore, by impairing mitochondrial integrity and mitophagy, the overabundance of Aβ and hyperphosphorylated tau proteins can exacerbate neurodegeneration in AD by increasing abnormal aggregation. Additionally, aberrant tau proteins and Aβ deposits can interfere with mitochondrial dynamics by dysregulating fission/fusion proteins, resulting in excessive mitochondrial fragmentation and eventual dysfunction [49]. Most recent studies suggest that APP-C99 accumulates in the ER’s MAM domains due to its affinity for cholesterol, which causes extracellular cholesterol to be taken up and transported from the plasma membrane to the ER. Consequently, MAM functioning undergoes constant turnover and becomes chronically elevated [50]. One study found that altered mitochondrial morphology in APP/PS1 mice is caused by changes in the expression of mitochondrial fission and fusion proteins. These changes are correlated with age, suggesting that aberrant mitochondrial dynamics may be an early indicator of AD progression [51]. A reduction in MFN2 at MAMs, along with other changes in mitochondrial dynamics, has been noted in a mouse neuroblastoma cell line overexpressing human APP with the familial Swedish mutation [52].

AD models show elevated mitofusin-2 (MFN2) expression and enhanced ER–mitochondria apposition, suggesting hyperactive MAM signaling [53]. Lipid metabolism is also altered: MAMs regulate phospholipid and cholesterol transfer, and their overactivation contributes to aberrant lipid profiles, including ceramide accumulation, which is known to exacerbate amyloid-beta (Aβ) production and tau hyperphosphorylation [54,55].

### 3.2. Parkinson’s Disease (PD)

PD is the second most common neurodegenerative condition after AD, affecting millions of people worldwide. Characteristic symptoms of the disease include the loss of dopaminergic neurons in the substantia nigra pars compacta of the ventral midbrain and the accumulation of intracytoplasmic fibrillary inclusions known as Lewy bodies, which are primarily composed of misfolded and aggregated protein α-synuclein (α-syn). Guardia-Laguarta et al. demonstrated that α-syn is present in MAMs in human and mouse brain tissue and cell lines, despite being primarily cytosolic [56]. α-synuclein, a hallmark protein in PD, localizes to MAMs and disrupts their function [57]. Overexpression or aggregation of α-synuclein impairs lipid metabolism and disrupts the VAPB–PTPIP51 complex, reducing calcium transfer and fragmenting mitochondrial networks [58]. MAM dysfunction in PD centers on impaired mitochondrial quality control and calcium dysregulation. Loss-of-function mutations in PINK1 and Parkin, two key regulators of mitophagy, reduce ER–mitochondria tethering and calcium exchange, thereby impairing mitochondrial ATP production [28,53,59]. These mutations hinder the ability of damaged mitochondria to signal for removal, leading to the accumulation of dysfunctional organelles and increased oxidative stress [60]. Previous studies have examined the effects of Parkin overexpression on ER–mitochondria crosstalk in relation to the regulation of two important cellular parameters: ATP generation and Ca^2+^ homeostasis [61]. Primary fibroblasts from PD patients with PARK2 mutations and Parkin knockout mice exhibited perturbations in MAMs; particularly, the ER and mitochondria were in proximity together, Ca^2+^ flow to the cytosol increased, and MFN2 (which is implicated in ER–mitochondria tethering) increased [62].

Another potential cause of MAM interference in Parkinson’s disease is the interaction between dopamine receptors (DRs) and Sig-1R. DRs are essential for numerous neurological functions, including motivation, cognition, memory, and motor control. In vitro and in vivo studies have demonstrated heterodimerization of Sig-1R with dopamine D1 receptor (D1R) and D2R [63]. In a mouse model of experimental Parkinsonism (intrastriatal 6-hydroxydopamine lesions), treatment with a selective Sig-1R agonist led to a gradual and significant improvement in motor function [64]. Furthermore, reduced MAM contacts in PD models are associated with impaired autophagosome formation, contributing to proteotoxic stress. These combined defects highlight how MAM dysfunction may accelerate dopaminergic neuron degeneration in PD.

### 3.3. Amyotrophic Lateral Sclerosis (ALS)

ALS is characterized by progressive motor neuron death, and growing evidence implicates defective MAM signaling in its pathogenesis [65]. Mutant forms of superoxide dismutase 1 (SOD1) and TDP-43 aberrantly localize to MAMs, disrupting ER–mitochondria contacts and impairing calcium homeostasis [66]. This disruption results in mitochondrial swelling, loss of membrane potential, and bioenergetic failure, hallmarks of motor neuron degeneration. Mitochondrial function and autophagy have been identified as key factors in the progression of ALS. Although Bax inhibitor 1 (BI1) has been linked to neurodegenerative disorders, the specific mechanisms remain uncertain. This study investigates the therapeutic effects of BI1 overexpression in ALS, both in vivo and in vitro, and finds that it can reduce SOD1G93A-induced apoptosis, nuclear damage, mitochondrial dysfunction, and motor neuron axonal degeneration. Simultaneously, BI1 extends the disease onset time and lifespan of ALS animals, improves motor function, and alleviates neuronal, muscle, and neuromuscular junction damage, among other effects [67].

Loss of SIGMAR1, a MAM-resident chaperone, has been identified in both familial and sporadic forms of ALS. SIGMAR1 stabilizes the IP3R–VDAC1 calcium channel complex and regulates the ER stress response [39]. Its dysfunction leads to persistent ER stress, abnormal calcium flux, and impaired autophagy. Additionally, ER–mitochondria disconnection in ALS models impairs lipid metabolism, particularly phospholipid and cholesterol biosynthesis, which are essential for axonal maintenance and membrane repair [68].

### 3.4. Huntington’s Disease (HD)

HD is an autosomal dominant neurodegenerative condition characterized by a traditional trifecta of symptoms consistent with mobility problems, mental difficulties, and cognitive loss [69]. Mutant huntingtin (mHTT) leads to the selective degeneration of medium spiny neurons in the striatum [70]. In HD, MAM dysfunction is closely linked to the effects of the mHTT protein [71]. mHTT interferes with ER–mitochondria tethering proteins, altering MAM structure and function [72]. Studies have shown reduced mitochondrial calcium uptake and abnormal mitochondrial morphology in HD models, suggesting weakened inter-organelle communication [73].

MAM-localized proteins such as MFN2 and the VAPB–PTPIP51 complex are disrupted in HD, contributing to mitochondrial fragmentation and impaired oxidative phosphorylation [20]. Lipid metabolism is also affected; HD brains show abnormal levels of ceramides and cholesterol, indicating dysfunctional lipid exchange at MAMs [74]. Furthermore, impaired autophagy, a key contributor to mHTT accumulation, is linked to defective MAM signaling [75]. Disruption of MAM-localized autophagy regulators such as Beclin-1 and FUNDC1 may contribute to the failure of neuronal proteostasis in HD [75].

**Table 1:** Summary of MAM-related molecular alterations in major neurodegenerative diseases.

Disease	Key Altered MAM Proteins/Pathways	Experimental Model	Observed Effects	References
AD	↑ Presenilin-1/2, ↑ APP cleavage via γ-secretase, ↑ MFN2, ↑ IP3R, ↑ VDAC1, ↑ GRP75, ↑ VAPB–PTPIP51	Presenilin-mutant cells, fibroblasts from familial AD patients	Tau hyperphosphorylation, mitochondrial dysfunction, ROS production, increased ER–mitochondria Ca^2+^ transfer, mitochondrial Ca^2+^ overload, lipid metabolism impairment, and impaired autophagy.	[76,77]
PD	↓ PINK1, ↓ Parkin, ↑ mitofusin (MFN) bridges, ↑ PERK–UPR pathway	Drosophila melanogaster (fruit fly) Human and mouse brain tissue + transfected cell lines	Inhibition of MFN–ER contacts or PERK signaling is neuroprotective, even in the presence of persistent mitochondrial malfunction. Defective mitochondria trigger ER stress through mitofusin-mediated ER–mitochondria contacts, which leads to PERK branch of UPR and neurodegeneration.	[78,79]
ALS	Mutant SOD1-G93A, ↑ ER–mitochondria contact sites, ↑ VDAC1, ↑ IP3R, altered mitochondrial Ca^2+^ uptake, ↑ CHOP and PERK (ER stress)	Patient-derived fibroblasts, neuronal cultures, and animal models (mice, Drosophila) from previously referenced studies on, e.g., mice (Mus musculus)	Mutant SOD1 accumulates at MAMs, disrupting Ca^2+^ homeostasis and enhancing ER stress. This leads to decreased mitochondrial activity and motor neuron degeneration, resulting in respiratory failure and muscular denervation.	[80,81]
Huntington’s Disease (HD)	mHTT impairs ER–mitochondrial connections, promoting mitochondrial fission	Human neuroblastoma cells, HdhQ111 mice: homozygous for mutant huntingtin with polyQ expansion	Altered Ca^+^ intake can lead to increased sensitivity and decreased buffering, as well as excessive mitochondrial fragmentation, defective trafficking, and bioenergetics.	[82,83]

## 4. MAMs and Lipid Metabolism in Neurodegeneration

MAMs are essential for the regulation of lipid biosynthesis, transfer, and distribution between the ER and mitochondria. MAMs consist of a region of the ER enriched in several lipid biosynthetic enzyme activities and are reversibly tethered to mitochondria [84]. As specialized microdomains enriched with lipid-synthesizing enzymes and lipid transfer proteins, MAMs coordinate the synthesis of phospholipids, cholesterol esters, and sphingolipids, which is essential for neuronal membrane stability, synaptic function, and intracellular signaling [85]. Disruption of these lipid metabolic processes at MAMs has been increasingly linked to the pathogenesis of neurodegenerative diseases, where altered lipid profiles are frequently observed [19].

Disruptions in MAMs cause neurodegeneration by interfering with disease-specific molecular pathways that control calcium signaling, lipid metabolism, and mitochondrial function [53]. Mutations in presenilin-1 (PS1) cause oxidative stress, neuronal apoptosis, and mitochondrial calcium overload in Alzheimer’s disease (AD) by interfering with ER–mitochondria calcium transfer through the IP3R–VDAC1 complex [86]. Furthermore, abnormal amyloid precursor protein (APP) processing at MAMs raises the production of β-amyloid close to mitochondria, which further compromises mitochondrial function. Aggregation of misfolded α-synuclein in Parkinson’s disease (PD) interferes with the VAPB–PTPIP51 tethering complex at MAMs, affecting ATP synthesis and calcium homeostasis [87]. Additionally, mitophagy and MAM-mediated mitochondrial quality control are impacted by mutations in PINK1 and Parkin [20]. While C9orf72 repeat expansions are connected to changed ER–mitochondria contacts in amyotrophic lateral sclerosis (ALS), pathogenic mutations in TDP-43 and FUS disrupt calcium signaling and mitochondrial bioenergetics by interfering with MAM-resident tethering proteins [88]. In ALS, there is a significant decrease in SIGMAR1, a crucial MAM chaperone protein, which is linked to worsened neurodegeneration and compromised mitochondrial function [89]. These molecular outcomes highlight the critical role of MAM dysfunction in neurodegenerative diseases and reinforce their potential as therapeutic targets.

### 4.1. Phospholipid and Cholesterol Transfer

Phospholipid synthesis is one of the principal functions of MAMs. Given the high concentration of the enzyme acyl-CoA:cholesterol acyltransferase (ACAT) in MAMs, it has been hypothesized that MAMs may be a site for the production of neutral lipids and cholesterol [90]. Specifically, the ER synthesizes phosphatidylserine (PS), which is transported to mitochondria via MAMs, where it is decarboxylated to phosphatidylethanolamine (PE) by mitochondrial phosphatidylserine decarboxylase [91]. PE is vital for maintaining mitochondrial membrane curvature, respiratory chain integrity, and apoptotic regulation [92]. A deficiency in PE can impair mitochondrial fusion and oxidative phosphorylation, thereby promoting neurodegeneration [93].

MAMs also mediate cholesterol transfer from the ER to mitochondria, where it is used for steroidogenesis and membrane composition [94]. The enzyme acyl-CoA:cholesterol acyltransferase 1 (ACAT1), enriched at MAMs, converts excess free cholesterol into cholesterol esters for storage [95]. Dysregulated cholesterol trafficking has been implicated in AD, where altered MAM activity enhances cholesterol and phospholipid synthesis, potentially contributing to amyloidogenic APP processing [19]. Increased MAM–mitochondrial tethering, observed in AD models, leads to excessive lipid transfer and disrupted membrane composition, affecting organelle function and protein trafficking [19].

### 4.2. Ceramide and Sphingolipid Dysregulation

For several years, studies on sphingolipids linked to neurodegenerative diseases focused on changes in glycosphingolipids, specifically gangliosides, sulfatides, and glycosylceramides (cerebrosides). Given that many of these glycolipids are components of myelin and tend to accumulate in lipid storage illnesses (sphingolipidoses) caused by enzyme defects in glycolipid metabolism, this focus is justified [96]. MAMs are also central to sphingolipid metabolism, particularly the synthesis and trafficking of ceramides, sphingomyelin, and glycosphingolipids [97]. Ceramides are bioactive lipids involved in apoptosis, autophagy, and inflammation, all of which are dysregulated in neurodegenerative diseases [98]. Enzymes such as ceramide synthases (CerS) and sphingomyelinases, found in the ER and MAM domains, catalyze the conversion of sphingosine to ceramide and regulate its cellular levels. Sphingomyelinases located at MAMs stimulate the production of ceramide from sphingomyelin, whereas enzymes such as ceramide synthases (CerS), which are essential to the ER and also found in MAM domains, catalyze the de novo and salvage conversion of sphingosine to ceramide. Together, these enzymes control ceramide levels and signaling [99].

Ceramide is one of the most important and fundamental types of sphingolipids. It is an essential precursor for most complex sphingolipids and a key component of the overall metabolism of sphingolipids. Ceramide can regulate vital biological functions such as apoptosis, senescence, differentiation, and cell growth. It also acts as a second messenger in a variety of signal transduction pathways, and is involved in immune system regulation and inflammatory responses [100]. Elevated ceramide concentrations have been reported in the brains of patients with AD, PD, and Huntington’s disease (HD), suggesting a shared mechanism of lipid-induced toxicity [101]. At the MAM, increased ceramide accumulation alters membrane fluidity and promotes mitochondrial outer membrane permeabilization, facilitating cytochrome c release and apoptosis [102]. Ceramide-induced ER stress can disrupt calcium homeostasis and further compromise neuronal viability [103].

**Figure 3:**
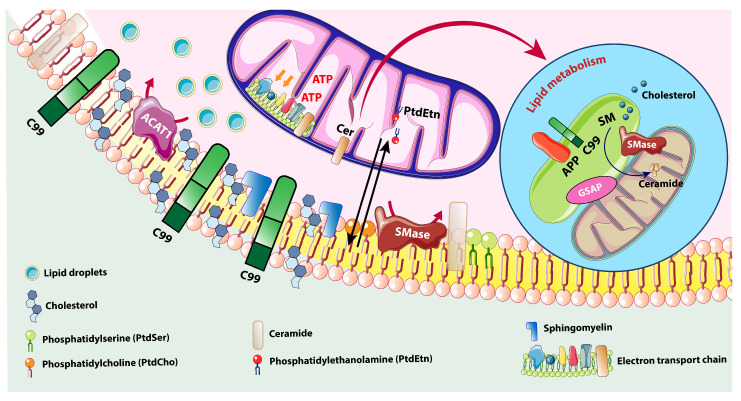
Lipid trafficking pathways at the mitochondria-associated membrane (MAM) interface and their dysregulation in AD and PD. Adapted from Agrawal et al., Neurobiol. Dis., 2020 [11], under the terms of the Creative Commons Attribution 4.0 International License (http://creativecommons.org/licenses/by/4.0/). Lipid trafficking pathways at the mitochondria-associated membrane (MAM) interface and their dysregulation in AD and PD. The illustration depicts the endoplasmic reticulum (ER, green), the MAM interface (beige), and the mitochondrion (orange), with lipid transfer routes indicated by arrows. Key lipid species, including phosphatidylserine (PS), phosphatidylethanolamine (PE), phosphatidylinositol (PI), and phosphatidylcholine (PC), are shown. Solid arrows represent normal trafficking routes across the MAM interface, and red arrows indicate altered lipid metabolism associated with AD/PD, such as decreased PS and mitochondrial PE and increased PI and PC. Tethering proteins facilitate close ER–mitochondria contacts, which are essential for lipid exchange and signaling. This figure highlights the crucial role of lipid homeostasis at the MAM and its disruption in neurodegenerative diseases [11].

In PD, abnormal accumulation of ceramides is associated with α-synuclein aggregation, and genetic mutations in glucocerebrosidase (GBA1), a key enzyme in sphingolipid metabolism, are among the strongest risk factors for developing PD [104]. Disruption of GBA1 impairs lysosomal degradation of glucosylceramide, leading to toxic buildup and enhanced MAM stress, creating a feedback loop of lipid dysregulation and neurotoxicity [105] (Figure 3).

### 4.3. Role in Lipid Droplet Formation and Turnover

MAMs also participate in lipid droplet (LD) biogenesis, acting as initiation sites for LD nucleation and expansion [106]. These droplets store neutral lipids like triglycerides and cholesterol esters, serving as reservoirs for cellular energy and membrane lipids during stress [107]. Although several hypotheses have been proposed for LD formation, the budding mechanism is the most commonly recognized. Enzymes such as MGAT1–3, DGAT1,2, and ACA1,2 participate in LD formation through neutral lipid synthesis of triglycerides and sterol esters, which includes pathways such as the monoglyceride and phosphatidic acid pathways [108]. When LDs interact with lysosomes, structural perilipin family members, perilipin 2 (PLIN2) and PLIN3, that encircle the LDs, can be broken down via chaperone-mediated autophagy (CMA) [109]. Proteins such as seipin, DGAT2, and PLIN2, which are implicated in LD formation, are enriched at ER–mitochondria contact sites, indicating that MAMs orchestrate the spatial organization of lipid storage.

In neurodegenerative diseases, impaired LD dynamics may exacerbate oxidative stress and lipid peroxidation. Excessive or persistent LD accumulation can signify metabolic dysfunction and is observed in AD, ALS, and HD models [110]. For instance, astrocytes in AD brain tissues show increased LDs, potentially reflecting compensatory responses to lipid imbalance and mitochondrial dysfunction. Furthermore, the failure to properly mobilize LDs due to MAM disruption compromises neuronal resilience under metabolic and oxidative stress conditions [111].

## 5. MAMs and Calcium Signaling

Calcium (Ca^2+^) signaling is fundamental to neuronal function, regulating synaptic activity, gene expression, mitochondrial metabolism, and apoptotic pathways. Mitochondria-associated membranes (MAMs) play a crucial role in orchestrating inter-organelle calcium exchange, particularly between the ER, the main intracellular calcium reservoir, and mitochondria, which transiently buffer calcium for metabolic and signaling needs [20]. Disruption of this tightly regulated calcium flux at MAMs significantly contributes to neurodegeneration, as it leads to mitochondrial calcium overload, oxidative stress, and energy failure. This section focuses on the molecular machinery mediating calcium transfer at MAMs, its dysregulation in disease, and the downstream consequences on mitochondrial bioenergetics [112].

### 5.1. Role of the IP3R–GRP75–VDAC1 Complex in Calcium Flux

The principal molecular machinery responsible for ER-to-mitochondria calcium transfer at MAMs is the IP3R-GRP75-VDAC1 complex, which is located at the contact sites between the ER and mitochondria [112]:

Inositol 1,4,5-trisphosphate receptor (IP3R) is a calcium channel on the ER membrane that releases Ca^2+^ into the cytosol upon activation [113].

Glucose-regulated protein 75 (GRP75) acts as a molecular chaperone, physically bridging IP3R on the ER with voltage-dependent anion channel 1 (VDAC1) on the outer mitochondrial membrane [114].

Once Ca^2+^ reaches the mitochondrial outer membrane through VDAC1, it is rapidly taken up by the mitochondrial calcium uniporter (MCU) complex on the inner mitochondrial membrane [115]. Electron tomography research has revealed specific contact zones between the mitochondria and the ER, divided by a small cytoplasmic gap of approximately 10–30 nm. These areas, referred to as MAMs, facilitate the direct transfer of calcium (Ca^2+^) from the ER to the mitochondria, avoiding the cytosol and reducing signaling delay or dilution. This close proximity promotes the formation of Ca^2+^ microdomains, ensuring rapid and localized signaling to support ATP synthesis and other mitochondrial processes. Furthermore, due to the close coupling at MAMs, mitochondria can affect ER Ca^2+^ homeostasis, creating a two-way communication channel that is essential for cellular energy metabolism and survival [6,116,117].

### 5.2. Dysregulated Calcium Transfer and Mitochondrial Overload

In healthy neurons, calcium flux through the IP3R-GRP75-VDAC1 complex is tightly regulated. However, in neurodegenerative diseases such as AD, PD, and ALS, this regulation is disrupted [37].

In AD, mutations in presenilin-1 and -2 enhance the sensitivity of IP3R, resulting in excessive calcium release from the ER. Combined with increased ER–mitochondria contacts, this leads to exaggerated calcium uptake by mitochondria, resulting in calcium overload. Similarly, in ALS, loss-of-function mutations in SIGMAR1, a chaperone that stabilizes IP3R and VDAC1 interactions, impair calcium buffering, destabilizing the entire transfer complex and leading to dysregulated calcium influx [118]. Excessive mitochondrial calcium uptake has several detrimental effects:

It opens the mitochondrial permeability transition pore (mPTP), resulting in membrane depolarization and the release of pro-apoptotic factors such as cytochrome c [119].

It promotes the generation of ROS, causing oxidative damage to proteins, lipids, and mitochondrial DNA [120,121].

It disrupts mitochondrial fission–fusion dynamics, promoting fragmentation and mitophagy [122].

These effects are particularly harmful to neurons, which have high energy demands and limited regenerative capacity.

Excessive mitochondrial calcium uptake can activate the mitochondrial permeability transition pore (mPTP), a large-conductance channel that causes membrane potential loss, swelling, outer membrane rupture, and cytochrome c release. This activates caspases and promotes apoptosis, which contributes to neurodegeneration [123].

### 5.3. Consequences for Mitochondrial Bioenergetics

Mitochondrial calcium plays a dual role in bioenergetics [41]. Under physiological conditions, low-to-moderate calcium levels in the mitochondrial matrix stimulate key dehydrogenases in the tricarboxylic acid (TCA) cycle, including pyruvate dehydrogenase, isocitrate dehydrogenase, and α-ketoglutarate dehydrogenase. This enhances NADH production and promotes oxidative phosphorylation (OXPHOS), thereby increasing ATP output [124,125,126,127,128].

In pathological states where calcium influx is excessive, mitochondrial function becomes impaired.

Overloaded mitochondria exhibit membrane depolarization, compromising the proton gradient necessary for ATP synthesis [129]. Elevated ROS levels oxidize respiratory chain complexes, impairing electron transport and further reducing ATP production [130]. Energy deficits lead to neuronal dysfunction, loss of synaptic transmission, and eventual apoptosis [131]. In neurodegenerative diseases, these mitochondrial bioenergetic impairments correlate with disease severity. For example, decreased mitochondrial membrane potential and ATP levels are consistently observed in neurons derived from AD and PD patients and animal models [132]. MAM dysfunction, through impaired calcium signaling, thus serves as a trigger for bioenergetic collapse and neurodegeneration [53].

## 6. MAMs in Regulation of Autophagy and Apoptosis

Mitochondria-associated membranes (MAMs) are more than structural connectors between the endoplasmic reticulum (ER) and mitochondria; they serve as active platforms that modulate cellular stress responses, particularly autophagy and apoptosis [133]. These processes are vital for maintaining neuronal health, particularly under conditions of oxidative stress, metabolic imbalance, or protein aggregation, all hallmarks of neurodegenerative diseases [134] (Table 2). The functional crosstalk between MAMs, autophagosome formation, ER stress signaling, and mitochondrial integrity positions them as central regulators of cell fate [7].

### 6.1. Crosstalk Between MAMs and Autophagosome Formation

The ER appears to play a critical role in the early stages of autophagosome formation. When autophagy is induced in mammalian cells, Atg proteins are recruited to the ER, where they attach to the expanding phagophore or isolation membrane between ER-associated membrane sheets known as the omegasome [135]. Autophagy is a conserved cellular degradation process that removes damaged organelles and misfolded proteins [136]. Emerging evidence suggests that MAMs serve as key sites for autophagosome biogenesis. Studies have shown that essential autophagy proteins, such as ATG14, Beclin-1, and VPS34, localize at MAMs during autophagy induction. This localization supports the hypothesis that the MAM microdomain provides the necessary lipids, curvature, and spatial proximity required to initiate autophagosome membrane nucleation [137].

FUNDC1, a MAM-anchored mitophagy receptor, further supports this model [138]. Under hypoxic or stress conditions, FUNDC1 recruits autophagy machinery to the MAM, facilitating the targeted removal of dysfunctional mitochondria via mitophagy [139]. FUNDC1 experiences post-translational changes during hypoxic stress, namely phosphorylation at Ser13 and dephosphorylation at Tyr18, which increase its binding affinity to LC3. The recruitment of autophagic machinery to MAMs, which facilitates mitochondrial turnover and selective mitophagy, depends on this interaction [140]. In neurodegenerative diseases, disruptions in MAM structure or tethering proteins such as MFN2 impair autophagosome formation and mitochondrial clearance, resulting in the accumulation of damaged organelles and aggregated proteins, as observed in Alzheimer’s, Parkinson’s, and Huntington’s disease models [22]. MFN2 has been identified as a transmembrane mitochondrial GTPase required for mitochondrial fusion [141]. Mutations in MFN2 cause Charcot–Marie–Tooth disease type 2A (CMT2A) neuropathy [142]. CMT2A is one of the most common mutations that cause CMT2 (OMIM 609260, accounting for approximately 35% of cases). CMT2A manifests as a severe motor neuropathy or a motor neuropathy accompanied by significant proprioception loss. In addition to its role in mitochondrial fusion, MFN2 regulates cell survival, cell proliferation, ER stress, and autophagy [143]. Taken together, MFN2 appears to play an important role in autophagosome formation, possibly by maintaining mitochondrial membranes and MAM integrity. This role is impaired in MFN2 deficiency and MFN2-induced CMT2A mutations, resulting in autophagy impairment and disruption of the balance between mitophagy and general autophagy [144].

Thus, MAM dysfunction may directly compromise autophagy efficiency, exacerbating proteotoxic and oxidative stress, two key drivers of neurodegeneration.

### 6.2. ER Stress, Unfolded Protein Response (UPR), and Mitochondrial Apoptosis

Disruption of ER protein folding, whether caused by exogenous or endogenous factors, induces a cellular stress response known as ER stress. ER stress aims to restore ER homeostasis through an integrated signaling mechanism known as the ER unfolded protein response (UPRER). In cases of severe toxic or prolonged ER stress, the UPRER’s pro-survival function is converted into a lethal signal, which is transmitted to and executed by mitochondria [145]. MAMs are critically involved in the coordination of ER stress signaling and mitochondrial apoptosis [7]. When misfolded proteins accumulate in the ER, cells activate the unfolded protein response (UPR) to restore homeostasis. However, persistent or excessive ER stress shifts the UPR from an adaptive to a pro-apoptotic mode [146]. MAMs are directly involved in this transition by modulating ER–mitochondria Ca^2+^ flux, ROS generation, and mitochondrial outer membrane permeabilization [26].

Proteins such as CHOP, ATF4, and IRE1α, which are central components of the UPR, are activated at or near MAMs. In parallel, the IP3R-GRP75-VDAC1 complex at MAMs facilitates mitochondrial calcium overload during unresolved ER stress, triggering the opening of the mitochondrial permeability transition pore (mPTP) [20]. This cascade results in the release of cytochrome c, activation of caspase-9 and -3, and ultimately, apoptosis. Bcl-2 family proteins are key regulators of mitochondrial apoptosis. Bax, a pro-apoptotic protein, is recruited to the outer mitochondrial membrane in response to cellular stress, promoting membrane permeabilization and cytochrome release. In contrast, Bcl-2, an anti-apoptotic member, binds to MAMs and regulates calcium flux from the ER to mitochondria, preventing calcium overload and apoptosis. The balance of Bax activation and Bcl-2 inhibition at MAMs is critical in determining cell fate during neurodegenerative stress [147]. This family includes both pro- (Bak, Bid, and Bax) and anti-apoptotic (Bcl-2, Bcl-XL, and A1/Bfl-1) members that work together to maintain mitochondrial membrane integrity [148].

### 6.3. MAMs in Cell Fate Decisions

Recent studies have revealed that death signals can be transmitted between the ER and mitochondria. MAMs are critical in the complex interplay between these two organelles. MAMs play critical roles in lipid synthesis, Ca^2+^ homeostasis, ER–mitochondrial coordination, and death signal transduction between the ER and mitochondria. Clarifying the structure and function of MAMs will provide new insights into the pathological mechanisms underlying neurodegenerative diseases, aging, and cancer. This review includes recent studies focusing on the structure and function of MAMs, as well as their roles in cell death, particularly apoptosis [7]. The balance between pro-survival autophagy and pro-death apoptosis is delicately regulated at the ER–mitochondria interface [149]. Under mild stress conditions, MAMs promote autophagic responses that remove damaged organelles and restore homeostasis. However, under prolonged or severe stress, the same MAMs may facilitate apoptotic signaling through calcium overload, mitochondrial dysfunction, and caspase activation [150].

The plasticity of MAMs is central to this dual functionality. Proteins such as MFN2, PTPIP51, and VAPB not only mediate ER–mitochondria contact but also influence the recruitment of autophagy or apoptosis effectors depending on cellular conditions. In neurons, which are particularly vulnerable to energy deficits and oxidative stress, even minor alterations in MAM function can shift the balance toward irreversible damage [25].

Alterations in lipid composition at MAMs, such as increased ceramide or cholesterol content, can modulate membrane curvature and signaling pathways involved in apoptosis. This lipid-mediated modulation reinforces the concept of MAMs as dynamic platforms where structural, metabolic, and signaling elements converge to determine neuronal survival.

**Table 2:** MAM-associated regulators of autophagy and apoptosis in neurodegenerative models.

MAM Component	Function	Impact on Cell Fate	Disease Context	References
IP3R-GRP75-VDAC1 Complex	Facilitates ER–mitochondrial Ca^2+^ transfer	Excessive Ca^2+^ causes mitochondrial depolarization and apoptosis.	AD, ALS	[80]
SIGMAR1	Stabilizes IP3R and regulates the ER stress response	Loss leads to ER stress, impaired Ca^2+^ signaling, and cell death.	ALS, HD	[151]
MFN2	ER–mitochondria tethering and mitochondrial fusion	MFN2 loss inhibits autophagosome formation and promotes apoptosis.	AD, PD	[152]
FUNDC1	Recruits LC3 to MAMs during hypoxia	Promotes mitochondrial quality control by facilitating mitophagy during stressful conditions (e.g., hypoxia), preventing the accumulation of damaged mitochondria.	PD	[139]

## 7. Therapeutic Potential of Targeting MAMs

**Figure 4:**
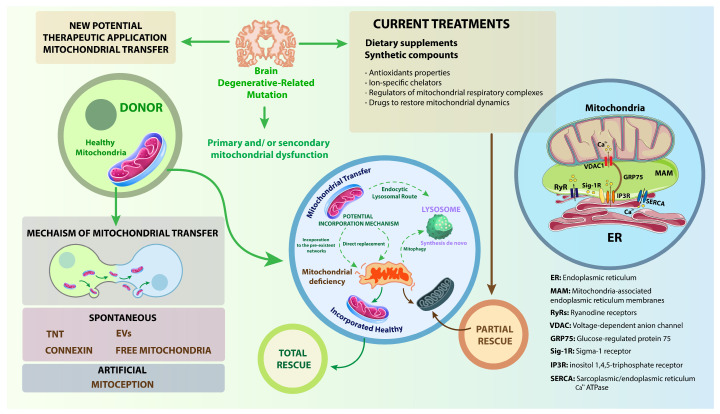
Potential therapeutic targets at MAMs and their proposed mechanisms of neuroprotection. Reproduced from Bustamante-Barrientos et al., [176] *Journal of Translational Medicine*, 2023 under the terms of the Creative Commons Attribution 4.0 International License (http://creativecommons.org/licenses/by/4.0/). Potential therapeutic targets at mitochondria-associated membranes (MAMs) and their proposed mechanisms of neuroprotection. This schematic illustrates the ER–MAM–mitochondria interface, highlighting three key proteins: MFN2 (mitofusin-2), PACS-2 (Phosphofurin Acidic Cluster Sorting Protein 2), and ATAD3A (ATPase Family AAA Domain-Containing Protein 3A). MFN2 promotes ER–mitochondria tethering, which supports calcium homeostasis and mitochondrial function. PACS-2 regulates autophagy, a critical process for cellular quality control. ATAD3A reduces oxidative stress and supports mitochondrial DNA integrity. All pathways converge on reducing oxidative stress and enhancing neuroprotective mechanisms, representing promising therapeutic strategies for neurodegenerative disorders. Schematic representation of the signaling pathway [176].

Given their central role in regulating mitochondrial function, lipid metabolism, calcium signaling, and cell fate decisions, MAMs have emerged as promising therapeutic targets in neurodegenerative diseases [19,153]. Disruption of MAM integrity is increasingly recognized as a converging point in disorders such as AD, PD, ALS, and HD [53,154]. Therapeutic approaches aimed at restoring MAM function or modulating MAM-resident proteins offer new avenues to correct underlying pathologies [13,36]. This section highlights key strategies being developed, including small molecules, natural compounds, and gene/protein modulation approaches targeting MAM components. MAMs represent a therapeutically tractable interface at the crossroads of metabolism, signaling, and cell survival. Targeting MAMs with small molecules, peptides, natural compounds, or gene therapy offers novel strategies for restoring cellular homeostasis in neurodegeneration [5] (Figure 4). As research advances, a more comprehensive understanding of MAM molecular dynamics will help develop more specific and effective interventions for modifying disease progression and improving neuronal resilience. Experimental evidence from model membranes shows that increased cholesterol or saturated lipid content reduces membrane fluidity, which lowers permeability to nonionic molecules such as urea and ammonia. Because MAMs are rich in such lipid species, changes in fluidity may have a significant impact on the efficiency of lipid transfer and calcium signaling between the ER and mitochondria [155].

### 7.1. Small Molecules and Peptides Targeting MAM Proteins

Several small molecules and peptide-based agents have shown promise in modulating MAM-associated proteins to restore ER–mitochondria communication and cellular homeostasis. A notable example is TAT-MP1Gly, a cell-permeable peptide that disrupts the interaction between PTPIP51 and VAPB, reducing excessive ER–mitochondria tethering in AD models and normalizing calcium homeostasis. By fine-tuning MAM contacts, such peptides can prevent mitochondrial calcium overload and oxidative stress [58,156]. The restoration of MAM integrity using small-molecule modulators has emerged as a promising therapeutic approach in neurodegenerative diseases such as Alzheimer’s disease [19]. Several pharmacologically active compounds, also known as “small modules,” have been shown to stabilize MAM structure and function by modulating lipid composition, improving membrane fluidity, and increasing protein-mediated tethering between the ER and mitochondria [157]. Resveratrol, a polyphenol that activates SIRT1 and improves mitochondrial biogenesis, has been shown to reduce ceramide accumulation at MAMs, restoring membrane fluidity and promoting efficient lipid and calcium exchange [158]. Similarly, curcumin’s antioxidant and lipid-modulating properties regulate cholesterol distribution and reduce oxidative stress at the MAM interface [159]. These small molecules not only repair damaged membrane architecture, but they also restore critical MAM-mediated processes like autophagy, ATP production, and apoptosis regulation [133].

Sigma-1 receptor (SIGMAR1) agonists, such as PRE-084, represent another class of MAM-targeted therapeutics [160]. SIGMAR1 resides at MAMs and plays a role in stabilizing IP3R-mediated calcium flux. The sigma-1 receptor (Sig-1R) is a chaperone found primarily at the mitochondrion-associated endoplasmic reticulum (ER) membrane (MAMs) and functions as a dynamic pluripotent modulator in living systems. At the MAM, Sig-1R regulates Ca^2+^ signaling between the ER and mitochondria and helps maintain the structural integrity of MAMs [161]. Pharmacological activation of SIGMAR1 improves mitochondrial function, enhances autophagy, and protects against ER stress in models of ALS and HD [162]. Similarly, ACAT1 (SOAT1), an important enzyme in cholesterol esterification, has been linked to lipid homeostasis and foam cell biology. Although ACAT1 inhibitors such as avasimibe were tested in clinical trials, they did not produce consistent cardiovascular benefits and, in some cases, increased risk. Although previous genetic studies suggested potential links to AD, genome-wide analyses have yet to confirm these findings [163].

These interventions highlight the feasibility of targeting specific MAM-resident proteins or protein–protein interactions as a therapeutic modality.

### 7.2. Natural Compounds (e.g., Curcumin and Resveratrol)

Natural compounds with antioxidant, anti-inflammatory, and neuroprotective properties have also been shown to influence MAM function [164]. Curcumin, a polyphenol derived from *Curcuma longa*, restores MAM integrity by reducing oxidative stress and modulating calcium flux [165]. In AD models, curcumin was found to attenuate mitochondrial dysfunction and reduce tau hyperphosphorylation, potentially through effects on ER–mitochondria signaling pathways [166].

Resveratrol, a stilbene found in grapes and red wine, activates SIRT1 and PGC-1α, enhancing mitochondrial biogenesis and function [167]. It also modulates autophagy and may restore MAM-dependent mitophagy in PD and ALS models [168]. Furthermore, resveratrol reduces ceramide levels and improves membrane fluidity, mitigating lipid-mediated stress at MAMs [169].

Other natural agents, such as quercetin, berberine, and epigallocatechin gallate (EGCG), are also being investigated for their ability to modulate ER stress, mitochondrial dynamics, and lipid metabolism (Table 3), all of which are regulated through MAMs [170]. Owing to their multitargeted actions, these compounds represent potential options for combinatorial therapy aimed at restoring MAM function.

**Table 3:** Clinical and preclinical studies of curcumin, resveratrol, and quercetin in neurodegeneration.

Compound Name	Doses	Title	Outcome Measure	NCT Number
Resveratrol, Quercetin, and Curcumin (RQC)	2000 mg curcumin/day, 334 mg resveratrol/day, 60 mg quercetin/day,	RQC for the Prevention of Alzheimer’s Disease and Retinal Amyloid-β	Change in retinal amyloid-β, progression to clinically relevant cognitive decline (MMSE)	NCT06470061
Resveratrol	2 capsules of 20 mg in the morning and in the evening (4 capsules in total/day = 80 mg/day) every day for 1 year	Resveratrol and Huntington Disease (REVHD)	Measurement of the rate of caudate atrophy before and after one year of treatment with resveratrol in patients with early-onset HD using volumetric MRI	NCT02336633
Curcumin	1 g/day curcumin	A Pilot Study of Curcumin and Ginkgo for Treating Alzheimer’s Disease	Change in isoprostane level in plasma	NCT00164749
	https://clinicaltrials.gov/ “URL (accessed on 9 July 2025)”.			

### 7.3. Gene Therapy and Protein Modulation Strategies

Advances in gene therapy have opened new possibilities for directly modulating MAM-associated proteins [171]. In various disease models, including neurodegeneration and metabolic disorders, gene therapy techniques employing adeno-associated viruses (AAVs) to deliver ER chaperones or UPR components have shown encouraging outcomes in improving proteostasis and reducing ER stress [172].

Mutations such as the pE102Q variant in the SIGMAR1 gene affect Sig-1R function in ALS, resulting in decreased mitochondrial ATP production, proteasome dysfunction, impaired ER–mitochondria communication, and increased neuronal susceptibility to ER stress. In ALS models, gene therapy approaches that target Sig-1R function restoration or enhancement, or modify its downstream pathways, offer a promising means of improving proteostasis, mitochondrial health, and overall neuronal survival [173]. In these models, SIGMAR1 gene therapy demonstrated neuroprotective effects by enhancing ER–mitochondria communication and reducing motor neuron loss [174]. Similarly, MFN2 gene delivery has been used to restore proper mitochondrial dynamics and calcium buffering in PD and optic atrophy models [175].

Gene and protein modulation strategies offer a highly targeted means of correcting MAM-associated dysfunction at the molecular level, particularly in monogenic or early-onset neurodegenerative disorders.

Drug development for AD remains challenging. While the amyloid cascade, tau hyperphosphorylation, and mitochondrial cascade hypotheses dominate, none fully explain AD pathogenesis or halt its progression. Emerging evidence highlights the role of mitochondria–ER contacts (MAMs) in early AD pathology, including Ca^2+^ dysregulation, mitochondrial dysfunction, oxidative stress, and impaired autophagy. MAMs regulate Ca^2+^ homeostasis through ER–cytoplasmic flux, with both excessive and reduced Ca^2+^ signaling contributing to disease. Modulation of MAM-resident enzymes (e.g., ACAT1) and MAM-C99 alters cholesterol metabolism, lipid homeostasis, amyloid synthesis, and synaptic transmission. MAMs also initiate autophagy, and the reduced expression of tethering proteins (Sig-1R, VAPB–PTPP51, and Mfn2–Mfn1) disrupts this process. These findings position MAMs as a critical target in AD pathogenesis.

## 8. Conclusions and Future Perspectives

MAMs play a crucial role in cellular metabolism by regulating calcium homeostasis, lipid metabolism, and mitochondrial function, thereby supporting overall metabolic processes. MAMs coordinate vital processes that affect the health and viability of neurons by physically and functionally linking the endoplasmic reticulum and mitochondria. The pathogenic cascades observed in AD, PD, ALS, and HD can be initiated and exacerbated by the dysregulation of MAMs, whether through abnormal protein–protein interactions, lipid imbalances, or disrupted calcium signaling. MAMs are particularly appealing targets because of their integrative function in neurodegeneration. MAMs serve as central signaling platforms where several pathological triggers converge, rather than being discrete contributors to pathology. They mediate the interactions between ER stress and energy failure, mitochondrial dysfunction and compromised proteostasis, and metabolic stress and programmed cell death. Therefore, they serve as both promising targets for therapeutic intervention and early markers of disease progression. To restore MAM integrity and function, preclinical models have demonstrated promise in modifying MAM-associated pathways through the use of small molecules, natural compounds, and gene therapy techniques. To better understand disease susceptibility and progression, future research should examine the differences in MAM remodeling across cell types, sexes, and aging stages. Developing precision treatments that target organelle communication may be made easier with a deeper mechanistic understanding of MAM regulation under neurodegenerative stress. The extent to which MAM changes in neurodegeneration are compensatory or causative remains unclear. Furthermore, additional research is needed to determine the exact mechanisms by which MAMs alter lipid signaling, autophagy, and apoptosis under various stressors. Future studies may identify new biomarkers and offer mechanistic insights by combining MAM biology with systems neuroscience and multi-omics techniques. The development of precision therapies that target organelle communication pathways and early diagnostic techniques may be facilitated by an understanding of how MAMs respond, or fail to respond, to neurodegenerative stressors. Ultimately, MAMs play a crucial role in determining the fate of neurons rather than merely being passive observers of neurodegeneration.
